# A Synopsis of Current Practice in the Diagnosis and Management of Patients with Turner Syndrome in Turkey: A Survey of 18 Pediatric Endocrinology Centers

**DOI:** 10.4274/jcrpe.0003

**Published:** 2018-07-31

**Authors:** Ahmet Uçar, Ayhan Abacı, Özgür Pirgon, Bumin Dündar, Filiz Tütüncüler, Gönül Çatlı, Ahmet Anık, Aylin Kılınç Uğurlu, Atilla Büyükgebiz, (Turner Study Group)

**Affiliations:** 1 University of Health Sciences, Şişli Hamidiye Etfal Training and Research Hospital, Clinic of Pediatric Endocrinology and Diabetes, İstanbul, Turkey; 2 Dokuz Eylül University Faculty of Medicine, Department of Pediatric Endocrinology and Diabetes, İzmir, Turkey; 3 Süleyman Demirel University Faculty of Medicine, Department of Pediatric Endocrinology and Diabetes, Isparta, Turkey; 4 İzmir Katip Çelebi University Faculty of Medicine, Department of Pediatric Endocrinology and Diabetes, İzmir, Turkey; 5 Trakya University Faculty of Medicine, Department of Pediatric Endocrinology and Diabetes, Edirne, Turkey; 6 Adnan Menderes University Faculty of Medicine, Department of Pediatric Endocrinology and Diabetes, Aydın, Turkey; 7 Gazi University Faculty of Medicine, Department of Pediatric Endocrinology and Diabetes, Ankara; 8 Private practice

**Keywords:** Turner syndrome, diagnosis, growth, puberty, oestrogen, oxandrolone, osteoporosis, adult transition, screening, cardiac magnetic resonance imaging, thoracic aorta

## Abstract

**Objective:**

A comprehensive survey was conducted to evaluate the shortcomings of clinical care in patients with Turner syndrome (TS) in Turkey.

**Methods:**

A structured questionnaire prepared by the Turner study group in Turkey, which covered relevant aspects of patient care in TS was sent to 44 pediatric endocrinology centers.

**Results:**

Eighteen centers (41%) responded to the questionnaire. In the majority of the centers, diagnostic genetic testing, screening for Y chromosomal material, protocols regarding the timing and posology of growth hormone (GH) and estrogen, thrombophilia screening, fertility information and screening for glucose intolerance, thyroid, and coeliac diseases in patients with TS were in line with the current consensus. Thirteen centers (72.2%) performed GH stimulation tests. Only four centers (22.2%) used oxandrolone in patients with TS with very short stature. The majority of the centers relied on bone age and breast development to assess estrogen adequacy, though together with variable combinations of oestrogen surrogates. Two centers (11.1%) reported performing serum estradiol measurements. Eight centers (44.4%) routinely conducted cardiac/thoracic aorta magnetic resonance imaging. Screening for hearing, dental and ophthalmologic problems were performed by thirteen (72.2%), six (33.3%) and ten (55.6%) centers, respectively. Psychiatric assessments were made by four centers (22.2%) at diagnosis, with only one center (5.6%) requiring annual reassessments.

**Conclusion:**

Although we found some conformity between the current consensus and practice of the participating centers in Turkey regarding TS, further improvements are mandatory in the multi-disciplinary approach to address co-morbidities, which if unrecognized, may be associated with reduced quality of life and even mortality.

## What is already known on this topic?

International consensus guidelines concerning the diagnosis, treatment and follow-up of patients with Turner syndrome have been reviewed and updated in the last few years.

## What this study adds?

This is the first study to document the shortcomings of current practice in diagnosis, treatment and follow-up of patients with Turner syndrome in Turkey.

## Introduction

Turner syndrome (TS) is the most common female sex chromosome disorder with an incidence of 1 in 2000 to 1 in 2500 live female births (1). It is caused by the complete or partial loss of a second sex chromosome during embryonic development with or without cell line mosaicism.

Individuals may be diagnosed at any age from fetal through to adult. TS may be suspected *in utero* as a result of screening for fetal abnormalities, in infancy by the presence of lymphedema, often associated with webbed neck and coarctation of the aorta, in childhood as a result of growth failure, in adolescence as a result of short stature with pubertal delay or in adulthood as a result of premature ovarian failure (2). Optimal care of this population should include proactive screening for co-existing medical conditions, including imaging for cardiac and renal anomalies, monitoring for obesity and hypertension, evaluation of developmental or psychoeducational abnormalities, hearing loss, autoimmune diseases and short stature. Ovarian dysfunction and a high probability of infertility should be anticipated (2).

Owing to recent advances in the diagnosis and management of patients with TS (3,4), the Turner Study Group in Turkey set out to establish state-of-the art care for Turkish patients with TS in a new consensus statement to include these internatioanally endorsed recommendations and guidelines. 

To this end, we sought to determine the current status in the diagnosis and management of patients with TS among Turkish endocrinologists.

## Methods

All pediatric endocrinology centers in Turkey were invited, via email, to respond to a questionnaire. An experienced pediatric endocrinologist from each center was asked to complete the questionnaire. The questionnaire was constructed by an experienced pediatric endocrinologist (Atilla Büyükgebiz) in the Turkish Turner Study Group. The questionnaire included multiple choice questions (with the option to include a comment by the respondent) on the following issues in sequence: demographics of the participating center, the diagnosis of TS, treatment for short stature, hormone replacement therapy, cardiac imaging, osteoporosis, fertility, adult transition and screening for co-existent medical conditions. The questionnaire is available at request as a supplementary file. Ethical approval was not needed owing to lack of involvement of patients or patient data.

### Statistical Analysis

The data were reported as frequency distributions when feasible.

## Results

Eighteen centers (11 university hospitals, five government-based education and research hospitals, and one state hospital; 41.1%) returned the questionnaire. The approximate number of patients with newly diagnosed TS per year by the attending centers was <5 patients/year in ten centers (55.6%), 5 to 10 patients/year in seven centers (38.9%), and >10 patients/year in one center (5.6%). The total number of patients with TS followed up by the centers was >30 patients in nine centers (50%), <10 patients in four centers (22.2%), between 10 to 20 patients in three centers (16.7%) and between 20 to 30 patients in one center (5.6%). 

All but one center reported further attempts to confirm the clinical suspicion of TS if standard karyotype analysis was reported as normal. The frequency distribution of the genetic tests used by the centers if the standard karyotype is normal is shown in Table 1. Thirteen centers (72.2%) asked the genetics lab for repeat karyotype analysis with 30 metaphase counts. Of these 13 centers, four (22.2%) also requested fluorescent *in situ *hybridization (FISH) analysis. One center (5.6%) ordered repeat karyotype analysis with 100 metaphase counts, and three centers (16.7%) exclusively ordered FISH analyses. 

Ten centers (55.6%) screened for Y chromosomal material only in the presence of virilization. Two centers (11.1%) routinely screened for Y chromosomal material, whereas six centers (33.3%) did not screen for Y chromosomal material. 

Eight centers (44.4%) performed gonadectomy when Y chromosomal material was detected (four centers at puberty, three centers in the postpubertal period and one center did not provide timing data). Three centers (16.7%) preferred cautioned follow-up in patients with TS with Y chromosomal material. 

Thirteen centers (72.2%) reported that due to legal procedures, they performed two growth hormone (GH) stimulation tests to establish the eligibility of patients to receive GH therapy gratis. Six centers (33.3%) reported an additonal diagnosis of GH deficiency. Two centers (11.1%) also assessed nocturnal GH secretion.

The frequency distribution of the timing of GH treatment in patients with TS is depicted in Table 2. The majority of the participating centers (n=14, 77.8%) started GH when the height of the patient deviated from the growth curve. Four centers (22.2%) began treatment when height fell below the 3^rd^ percentile. Two centers (11.1%) started GH at diagnosis if the patient was aged from 4-12 years or older. 

The doses and dosing schedules of GH in patients with TS attending the participating centers are shown in Table 3. Nine centers (50%) started GH at a dose of 0.375 mg/kg/week and adjusted the dose depending on the clinical response of the patient. Of these 9 centers, three centers (16.7%) also indicated that they adopted a fixed dosing protocol. The other centers (n=9, 50%) adopted an initial GH dose of 0.045 mg/kg/day and adjusted the GH dose depending on the growth response and/or serum insulin-like growth factor 1 (IGF1) level. 

The majority of centers (n=14, 77.8%) did not use oxandrolone to improve final height in patients with TS. Only four centers (22.2%) adopted oxandrolone use, one of which also emphasized variable availability of the medication in Turkey.

The frequency distribution of the timing of estrogen treatment for induction of pubertal development is shown in Table 4. Seven centers (38.9%) started estrogen therapy at the age of 12 to 13 years, regardless of the age at initiation of GH treatment. Of these, one center indicated that they also had adopted a protocol involving commencement of estrogen at the age of 15 years, if GH was started after the age of 11 years. Three centers (16.7%) unconditionally withheld estradiol replacement until the age of 15 years, if GH was started after the age of 11 years. The timing of the start of estrogen therapy was dependent on the timing of initiation of GH treatment in four centers (22.2%). Six centers (33.3%) waited until the age of 13 years just in case spontaneous puberty occurred before commencing estrogen. Four centers (22.2%) also indicated that they did not favor waiting until the age of 15 years to start estrogen.

Only three (17%) centers used a routine screening protocol for potential thrombophilia, prior to oestrogen treatment. The remainder stated that they had asked the health care providers of the patient to provide information regarding a family history of thrombophilia.

Eleven (61.1%) centers used transdermal estrogen. Of these, four centers also used oral estrogen. Five centers (33.3%) exclusively used oral estrogen and one center used ethinyl estradiol. None of the centers used conjugated estrogen. One center did not specify the type of estrogen used. 

The surrogates of estrogen adequacy adopted by the centers are shown in Table 5. A variable combination of the surrogates was adopted by 16 centers (89%) to monitor estrogen adequacy. Breast development, according to Tanner staging (n=17, 94%) and bone age (n=16, 89%) were the most frequently selected estrogen surrogates. Two centers (11%) relied exclusively on the degree of breast development to assess estrogen adequacy. However only two centers (11%) directly monitored serum estradiol levels in addition to monitoring several surrogates of estrogen effect. 

Progesterone treatment by the centers was also subject to multiple responses, but the majority of centers awaited occurrence of withdrawal bleeding (n=11, 61%). Nine centers (50%) added progesterone when Tanner’s breast stage of 3 or 4 was attained. Three centers (17%) added progesterone after either one (n=1) or two years (n=2) of estrogen treatment.

Eight centers (44.4%) routinely scheduled patients for cardiac/thoracic aorta MRI either at diagnosis of TS or when they reached an age when general anesthesia was not required. Three centers (17%) ordered cardiac MRI if there was a clinical suspicion of cardiac abnormality. Seven centers (39%) did not routinely order cardiac/thoracic aorta MRI in patients with TS.

All but two centers (89%) reported routine screening of patients with TS for osteoporosis. Ten centers (56%) started evaluation for osteoporosis at age 15 years and over. 

Five centers (39%) exclusively informed parents and later the patients that infertility in TS was an expected end-point. Six centers (33%) performed follow-up with serum anti-mullerian hormone (AMH) levels as a means of monitoring the ovarian reserves of the patients. Eleven centers (61%) informed the patients and parents about the possibility of ovarian cryopreservation.

Fifteen centers (83%) transferred patients with TS to adult endocrinology outpatient clinics with a medical report and/or a phone call to the adult endocrinologist. Only two centers (11%) conducted an adult transition outpatient clinic. One center (5.6%) sent a copy of the patient’s medical file to the adult endocrinology clinic.

The distributions of the timing and screening frequencies of the parameters regarding co-existent complications in patients with TS are shown in Table 6.

## Discussion

This survey of 18 pediatric endocrinology centers in Turkey confirmed the need to provide updates to physicians using structured protocols reflecting the current international consensus in order to provide optimal care to patients with TS. While conformity was found among the centers regarding genetic tests to diagnose TS, GH use and hormone replacement therapy in TS, there were apparent discrepancies between centers and the current consensus regarding healthcare-related surveillance issues. We believe that some of these discrepancies were probably due to the variable availability of medical resources/equipment in different parts of Turkey.

In line with the suggestions of the American College of Medical Genetics (5), almost all the centers performed either karyotype analyses with 30 metaphase counts or FISH analyses when the index of clinical suspicion for TS was high in a patient but was not supported by standard karyotype analyses. However, the mean age at diagnosis of TS was 10.2 years in a recent national study examining the clinical characteristics of patients with TS in Turkey (6). Early diagnosis of TS is pivotal for improving preventive measures and treatment. To this end, there is ongoing promising research for early diagnosis of TS via neonatal screening with whole exome sequencing (7) or assessment of X chromosome, inactivation-specific, differentially methylated CpG sites (8). Such a screening method would be extremely beneficial for earlier diagnosis of TS in Turkey given the current mean age at diagnosis. The feasibility of this form of screening is one of the current areas of focus for the Turner Syndrome Study Group in Turkey, along with increasing awareness of TS among health care professionals, and to include TS in the differential diagnosis for girls with short stature, when baseline work-up does not yield definitive cause. 

More than half of the centers in the current survey attempted further analysis to investigate for Y chromosomal material should they find evidence of virilization in patients with TS, whereas one third of the centers did not. Y chromosome sequences occur in approximately 6% to 11% of patients with TS, which is of concern because approximately 10% of these go on to develop gonadoblastoma (9). Due to the risk of malignancy, many TS specialists recommend prophylactic gonadectomy (10) and cryptic Y material should especially be assessed in TS patients with virilization, even in the absence of a marker or ring chromosome (11). A recent study reported comparable rates of gonadoblastoma between patients with cryptic Y chromosome and patients with overt Y chromosome and recommended routine molecular screening for Y chromosome material for all patients with TS (12). The current guidelines recommend prophylactic gonadectomy in all patients with TS with Y chromosome identified on standard karyotyping (4). Molecular screening detection of Y chromosome sequences is currently recommended in individuals with TS with virilization but negative cytogenetic analyses and negative FISH. Gravholt et al (9) reported that careful follow-up with close observation of the gonads using ultrasonography could be an option in some patients with TS who harbour Y chromosomal material emphasizing that in most of these patients, malignancy does not occur. Overall, these controversial reports indicate the need for further studies to reach hard end-point conclusions on gonadectomy in patients with TS who are positive for Y chromosomal material. 

Despite the fact that current guidelines and reviews (3,4) do not recommend GH stimulation tests for patients with TS. However, 72% of the centers in the current survey conducted GH stimulation tests routinely because the high cost of GH treatment would then be covered by Turkish social security. Moreover, 33% of the patients were given GH with an inappropriate diagnosis of GH deficiency if they were found to have reduced response to GH stimulation tests in two tests. Although there is some evidence of heterogeneity regarding the GH IGF1 axis in patients with TS (13), current guidelines (3,4) suggest that GH should be started in patients with TS without the need for GH stimulation tests. There is ongoing collaboration between the Turkish Pediatric Endocrinology and Diabetes Society and the Turkish Ministry of Health to solve this issue. 

The majority of the centers in the current survey chose to start GH treatment when the patient with TS had evidence of growth failure, i.e., a height velocity <50^th^ percentile observed over six months, the child was already short or had a high likelihood of short stature, which is in line with the recommendation from the current guidelines (3,4). The optimal age to start GH has yet to be established.

In the current survey, the dosage of GH practised by the centers was around 45 to 53 µg/kg/day, which is closer to the lower range of the doses suggested by current guidelines (3,4). The Nordinet International Outcome study, which was conducted between 2006 and 2016, described real-life dosing patterns in children using GH owing to various pathologies including TS. In the Nordinet study, GH doses in patients with TS were found to have a tendency to be at the lower end of the recommendations of the practice guidelines and label ranges, albeit factors associated with this tendency were unclear (14). The reasons for avoiding higher doses need to be explored because there is a limited time for potential efficacy of GH in patients with TS owing to the delayed diagnosis of TS in Turkey (6).

Only four centers (22.2%) used oxandrolone in patients with TS in the current survey. If the diagnosis of TS and subsequent GH treatment is delayed, and/or adult height outcome data is likely to be unsatisfactory with the standard GH dose alone, the current consensus (3) recommends treatment with oxandrolone. The uncommon use of oxandrolone in Turkey, despite considerably delayed diagnosis of TS, might be due to the intermittent availability of the drug in Turkey, as in other parts of Europe.

Regarding estrogen replacement for induction of puberty, almost 40% of the centers in the current survey started estrogen treatment at age 12-13 years, and 20% of the centers delayed estrogen until 15 years if GH was started after the age of 11. Although there are concerns for compromised height potential in patients with TS older than 11 years who are GH treatment-naive, if estrogen is started around the age of 12 to 13, delay in estrogen replacement was associated with very poor outcomes regarding bone mineral density (BMD) measurements (15). The current consensus (4) does not recommend delaying estrogen in patients with onset of GH treatment after the age of 11, and favors estrogen replacement initiation around 11 to 12 years. The optimal approach to feminization for patients with TS in terms of estrogen formulation and estrogen dose progression is not clear. Yet, the guideline supports the practice of incremental dose increases approximately every six months to mimic the normal pubertal tempo until adult dosing is reached over a two to three-year period. The use of low-dose estrogen in prepubertal ages is still under investigation and is discouraged in the current guidelines (4). 

Apart from three centers (16.7%) in the current survey, profiling of the coagulation system prior to estrogen treatment was uncommon in our survey despite some evidence of abnormalities in coagulation in patients with TS (16). The current consensus does not recommend routine profiling of the coagulation system, but it advises investigating for a family history of coagulopathy. 

The route of estrogen administration has been a hot topic of research. About 60% of the centers in the current survey preferred transdermal estrogen over the oral form, in accordance with the recent consensus (4). Transdermal estrogen has been preferred over the oral route owing to its more physiological form and avoidance of the liver first-pass metabolism of oral estrogen (17,18). In a 2006 survey among physicians from the United States of America, 78% were found to prescribe conjugated estrogens (19), whereas in a European survey, 39% of the physicians were using ethinyl estradiol, 32% used oral micronized estradiol, and only 12% used conjugated equine estrogen (20). Only 8% to 10% of the physicians were found to prescribe transdermal estradiol (20). 

Clinical assessment, patient satisfaction, patient age and residual growth potential were considered as primary determinants of estrogen adequacy in the current consensus (4). Except for two centers, which exclusively relied on the degree of breast development as a criterion to assess estrogen adequacy, the centers in the current survey chose various combinations of surrogates of estrogen effect. The variables most commonly used by the centers were breast development in conjunction with bone age. Only two centers selected monitoring serum estradiol as a surrogate parameter to judge estrogen adequacy. In fact, serum estradiol measurement using an ultrasensitive assay may allow for titrating dosage. A protocol involving the use of transdermal estradiol (E2) and monitoring with an utrasensitive E2 assay does exist and is based on excellent studies (21,22), although estradiol concentrations that achieve maximal growth probably need further exploration.

The referral of patients with TS for baseline cardiac evaluation is well established in Turkey, as suggested in the guidelines. Patients with TS have a predilection for aortic dissection, which is almost six times more common than in the general population (15). Several indices of the aorta such as aortic size index as assessed through cardiac/thoracic MRI of the aorta are commonly used to predict possible occurrence of such a risk (23). However, MRI studies are expensive and carry additional risk in patients aged less than 12 years with a frequent need for deep sedation. Accordingly, several surrogate markers for dilation of the aorta and vascular disease are currently under investigation (24). Unfortunately, the present survey showed that only 44% of centers routinely performed cardiac/thoracic aorta MRI, either at diagnosis or at an age when anesthesia was feasible, which is concordant with the current consensus. In our opinion, this is one of the aspects of care for girls with TS in Turkey that requires particular attention and improvement because aortic dissections have been reported as early as age 4 years (25). Our survey did not explore the reasons for the significant lack of referrals for cardiac/thoracic aorta MRI, but the high cost of the procedure and the lack of availability of the required device in many centers could possibly be potential causes.

More than half of centers in the current survey routinely evaluated patients with TS for osteoporosis at age 15 yrs and over. The current guidelines (4) suggest screening patients with TS with DEXA scan after adult hormone replacement has been instituted, with moderate levels of evidence. Moreover, beginning at age 9 to 11 years, and then repeating every 2 to 3 years, serum 25 hydroxyvitamin D level measurements are recommended, despite low levels of evidence. The degree of vitamin D sufficiency in Turkish girls with TS is unknown as levels were not investigated in the current survey. Endogeneous and exogeneous estrogen exposure is associated with improved BMD, although women with TS and normal BMD still have increased fracture risk compared with controls (24). Results regarding BMD in patients with TS are not consistent owing to differences in methodologies and small bone size (4). Most females with TS have normal BMD. 

Fertility is one of the major concerns of patients and families affected by TS. In the current survey, around 60% of the centers informed the parents and the patients regarding the possibility of ovarian cryopreservation. Cryopreservation of mature oocytes and embryos is a proven fertility preservation approach, and cryopreservation of ovarian tissue is a promising technique with a growing number of live births, but is still at the investigation stage. Oocyte cryopreservation has been performed in children with TS aged as young as 13 years (3,4). However, the efficacy of the procedure needs to be proven on a larger scale. About 30% of the centers in the current survey reported that they regularly checked AMH levels in an effort to predict ovarian function. AMH was shown to be effective as a predictor of absent puberty, as AMH ≤2 SD for age predicted failure to enter puberty in young girls with TS and imminent primary ovarian insufficiency in adolescent and adult patients with TS (26,27). The use of AMH as a screening tool for ovarian function was not recommended in the current consensus. However, the use of AMH in conjunction with follicle-stimulating hormone in the context of fertility issues was discussed. 

Unfortunately, only two centers (11.1%) in the current survey held an adult transition outpatient clinic and more than 80% of the centers in the survey sent young patients with TS to the adult clinic with only a medical review report. Failures during the transitional phase to adult care may result in moderate healthcare outcomes and decreased quality of life. To be of help in overcoming problems at transition, starting at the age of 11 to 13 years, physicians should repeat information directly to the girl about medical and health issues that were disclosed to the parents (28). The process of transition readiness needs to be followed by the health care provider, possibly with the use of questionnaires enquiring about healthcare autonomy, self-care and disease management as has been described previously (28). Unfortunately, it is difficult to find experts in adult care. In adult life, less than 4% of patients with TS undergo all the recommended medical investigations on a regular basis (29). Many reasons have been described for transition failures such as poor self-advocacy or self-management, little family support or unsatisfactory cooperation between healthcare professionals and organizational structures (30,31,32,33).

Our survey revealed that co-morbidity screening in patients with TS needs to be improved because there were significant deviations from what was suggested in the current consensus (4). The screening of the TS patients by blood pressure measurements, metabolic parameters such as fasting plasma glucose, hemoglobin A1c, lipids, thyroid function tests, coeliac antibodies, liver transaminases and renal ultrasound evaluation were somewhat in conformity with the current consensus. However, the survey results displayed significant discordance for audiology, dental, ophthalmology, orthopedic and psychiatry consultations. 

Hearing loss is a well-known problem in patients with TS (34). Evaluation is indicated in all girls with TS at the time of diagnosis and at 2- to 5-year intervals (3,4). However, although more than 70% of the centers referred the patients for audiometric evaluation at diagnosis, follow-up referrals decreased to as low as 22% of the centers. In the socially- and bone-disadvantaged TS population, addressing hearing problems is of paramount importance. Considering the well-established increase in accidental falls in people who require hearing aids and the increased incidence of bone mineral abnormalities in TS, the importance of regular audiometric evaluation cannot be ignored.

Orthopedic, ophthalmologic, and dental evaluations were performed either at diagnosis in 11%, 56% and 33% of the centers, respectively, or as clinically indicated during the course of follow-up. It is recommended by TS experts that all these evaluations should be repeated at regular intervals (2). 

In the current survey, it is unfortunate that psychiatric evaluation of patients with TS was found to be among one of the aspects of care that was most lacking. Only 22% of the centers in the current survey referred patients with TS to the psychiatry outpatient clinic and of these, only one center made annual referrals. A full discussion of the psychosocial development in patients with TS is beyond the scope of this manuscript, there are known to be deficits in mathematical abilities, visuospatial processing and verbal skills which may worsen over time so that annual developmental and behavioral scales are recommended (28). A genetic basis rather than phenotype-related shortcomings perceived by patients with TS seems to be emerging as a major cause of the psychiatric alterations (35).

### Study Limitations

Our survey was not designed to delineate the factors associated with shortcomings of clinical care in patients with TS in Turkey. Further studies are needed to delineate these factors.

## Conclusion

The current survey revealed that issues regarding the diagnosis of TS, treatment of short stature, pubertal management, and fertility-related questions by the patients/ parents were addressed by many pediatric endocrinologists akin to those of Western countries. Yet, we also identified several shortcomings of care for patients with TS in Turkey when compared with developed countries, issues that are among the current areas of focus by the Turkish Turner Study Group.

## Figures and Tables

**Table 1:**
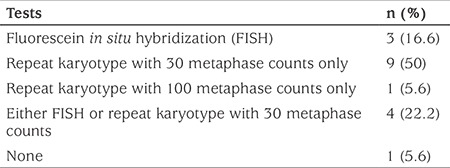
Frequencies of the genetic tests applied by the 18 pediatric endocrinology centers in patients clinically suspected as having Turner syndrome but with normal standard karyotype analyses

**Table 2:**
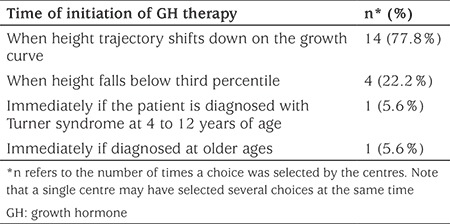
Frequency distribution of the criteria to start growth hormone in treatment-naive patients with Turner syndrome among 18 pediatric endocrinology centers in Turkey

**Table 3:**
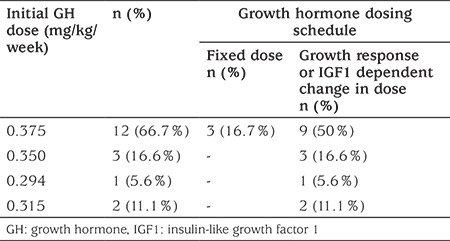
Frequencies of the initial dose and dosing schedule of GH in patients with Turner syndrome among 18 pediatric endocrinology centers in Turkey

**Table 4:**
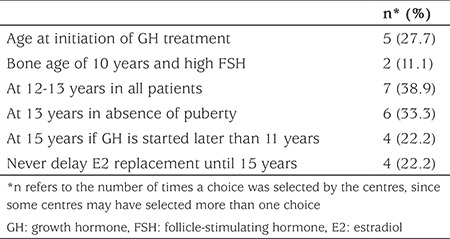
Frequency of the timing of estrogen treatment in patients with Turner syndrome among 18 pediatric endocrinology centers in Turkey

**Table 5:**
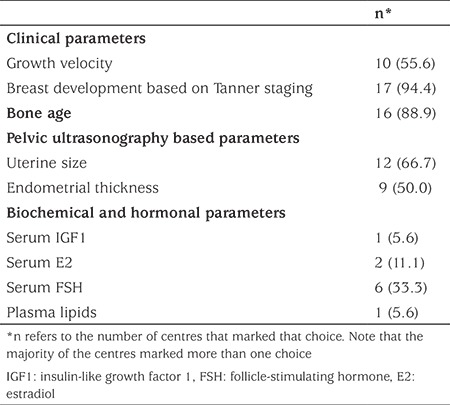
Frequency distribution of the parameters used by 18 paediatric endocrinology centers in Turkey to assess the adequacy of oestrogen replacement in patients with Turner syndrome

**Table 6:**
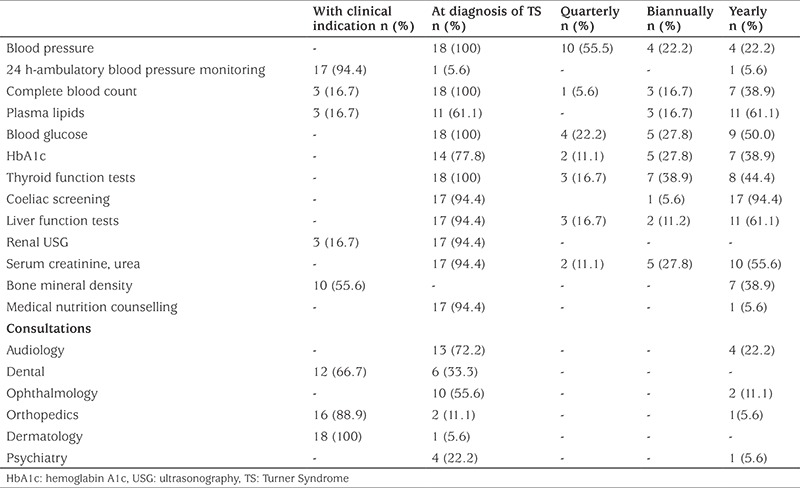
The distribution of the timing and screening frequencies of the parameters regarding co-existent complications in patients with Turner syndrome as assessed by 18 pediatric endocrinology centers in Turkey
